# Active Ageing: Mapping of Scientific Coverage

**DOI:** 10.3390/ijerph15122727

**Published:** 2018-12-03

**Authors:** José Álvarez-García, Amador Durán-Sánchez, María de la Cruz del Río-Rama, Diego Fernando García-Vélez

**Affiliations:** 1 Financial Economy and Accounting Department, Faculty of Business, Finance and Tourism, University of Extremadura, 10071 Cáceres, Spain; pepealvarez@unex.es; 2 Faculty of Business, Finance and Tourism, University of Extremadura, 10071 Cáceres, Spain; ads_1975@hotmail.com; 3 Business Organisation and Marketing Department, Faculty of Business Administration and Tourism, University of Vigo, 32004 Ourense, Spain; 4 Department of Economics, Universidad Técnica Particular de Loja (UTPL), Loja 11-01-608, Ecuador; dfgarciax@utpl.edu.ec

**Keywords:** Active Ageing, Active Ageing Paradigm, Bibliometric Study, International Databases of Bibliographic References

## Abstract

Population ageing is one of humanity’s greatest achievements with the elderly who offer valuable resources and make an important contribution to the structure of our societies. At the same time, this ageing population poses great challenges, as it requires greater economic and social needs. Institutions such as the World Health Organization (WHO) are promoting policies that aim at promoting active ageing, which is understood as the process of optimizing health, participation and security opportunities in order to improve people’s quality of life as they get older. The main objective of this study is to identify scientific production related to the area of Active Ageing. The work methodology used is the bibliometric analysis of the articles indexed in the multidisciplinary databases WoS and Scopus. There were 171 articles in WoS and 234 in Scopus that were selected, with a time limit in 2017. In the analysis carried out it is observed that active ageing is a topic that has aroused interest among researchers in recent years, proof of this is the increase both in the number of articles published in scientific journals and in the citations received. The Scopus database presents a greater coverage of the subject. The Overlap Index shows that Scopus covers 90.06% of the WoS articles and its Single Documents index is 34.19% versus 9.94% of WoS.

## 1. Introduction

Due to health care advances, an increase in per capita income, and the improvement of welfare and social standards, life expectancy has increased dramatically in the last decades [1,2]. Ageing is a natural process that involves physical, psychological, and social changes, and is a global phenomenon that requires coordinated actions at international, national, regional, and local levels (Governance of Ageing), which imply profound economic and social restructuring. In this sense, according to Walker [3] “active ageing is established as the leading global policy strategy in response to population ageing”.

**Figure 1:**
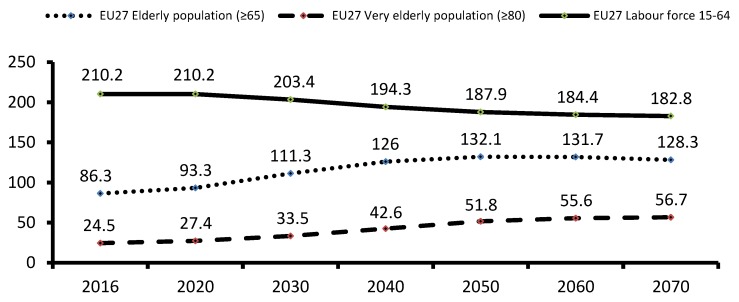
Forecast of the evolution of the elderly population and active population in EU-27 (in millions of people). Source: Own elaboration based on data from “The 2018 Ageing Report” [5].

An important aspect to be mentioned is that we are witnessing a change, which has been denominated the “Potential Support Ratio”, that is, the number of people aged between 15 and 64 for every person over 65 (an expression of the relationship between people of working age and people of non-working age) [4]. In 2016, the Potential Support Ratio of the EU-27 was 2.44, a figure which is expected to decrease to 1.83 in 2030, and even to 1.4 in 2050 (see Figure 1), with obvious adverse consequences for social security schemes. In an increasingly interconnected world, if the requirement that these demographic changes involve is not addressed in a rational way, there will be adverse socio-economic and political consequences in the not-so-distant future.

Many factors contribute to the longevity and dynamic process of active ageing: genetic characteristics (according to Herskind et al. [6]; Kaplanis et al. [7]; Murabito et al. [8], the genetics cause does not reach more than 25% of what each individual will be), gender, behavior, socioeconomic class, culture and environment [9]. That is, ageing is not only a biological process, but a process determined by a series of biological, social, psychological, and ecological factors. As a consequence, there is a great diversity of profiles among the elderly with a wide range of needs.

This is also seen by World Health Organization (WHO) [10], which developed the Model of the Determinants of Ageing, which reflects the influence of various factors that affect the ageing process and impact on active ageing. There are external factors: cross-cutting determinants (culture and gender), determinants related to health services and social services, determinants related to the physical environment (adapted physical environments), determinants related to the social environment (social support, opportunities for education and learning, etc.), economic determinants that influence people’s health, security and opportunities to participate actively in society. And on the other hand, there are Personal Behavioral Factors: behavioral determinants, determinants related to personal and psychological factors [11].

In this context, in recent decades, there has been a growing interest, both from scientific and political spheres, focused on developing and finding formulas that allow for ‘good ageing’, with the aim of providing greater autonomy to the elderly and social inclusion [9].

In this sense, there is no consensus on which activities are part of an active life in old age: physical activity, work activity, participation in economic, social, spiritual, civic and cultural affairs. The literature emphasizes more on considering a few activities for active ageing, which have an important socioeconomic impact [12], not considering psychological and social aspects of great importance. Some authors consider that leisure activities should also be included [13,14,15,16], learning activities [17], care of other family contexts [18], volunteer activities [19], and finally political participation [20,21,22,23,24,25]. Different classifications of active ageing activities can be seen in Burr et al. [26] and Villar and Celdrán [27], that use different classification criteria such as the degree to which a contribution for others is assumed, the degree of commitment, the degree of effort required, the resources necessary to carry it out, type of orientation (primarily social or individual), etc.

The World Health Organization (WHO) defined active and healthy ageing as the process of optimizing health, participation, and security opportunities to improve people’s quality of life as they get older [10]. This process aims at increasing individuals’ average quality of life and, at the same time, reducing the expenses in public pensions and medical treatment, as well as reducing the expenses related to the social policies of dependency.

The importance that everything related to active ageing has acquired promotes the publication of an extensive scientific literature on the subject in several areas of knowledge: psychology, pedagogy, gerontology, etc. Therefore, it is necessary to analyze the repercussion that these publications have within the academic field, which will help in developing new studies and identifying new directions and challenges for the future [28]. 

Several authors maintain bibliographic reviews to provide a global view of the coverage of research on the subject under study and synthesizing existing knowledge [29], however, it is not the only tool available for this. Within bibliometric research, the bibliographic databases are configured as a necessary tool. In the databases, scientific and technical publications are collected and stored in an organized manner, allowing the extraction of the documents in order to evaluate the performance of scientific activity and identify trends in research. Therefore, the validity of the work will depend on its proper selection [30]. 

Thus, the fundamental objective of this study is to perform an analysis of published scientific literature related to Active Ageing to determine the extent to which the concept of ‘active ageing’ has received scientific coverage, given its importance to guiding policy and practice in an ageing society. A bibliometric analysis of the indexed publications on this subject was carried out in the international databases WoS and Scopus. In addition, an analysis of coverage and overlap between the databases was carried out. With the purpose of locating documents focused on Active Ageing indexed in both databases, an advanced search for terms was carried out. There were 171 articles in WoS and 234 in Scopus that were identified and extracted. These documents make up the empirical ad hoc basis of the study.

The paper is structured as follows. After the introduction, the evolution of the concept of active ageing is described, and the pillars on which the current concept is based are defined. Next, in Section 3, the methodology of the study is described (databases, search strategy, calculations). Section 4 details the results obtained by applying the bibliometric indicators to the identified scientific production on active ageing. This section is completed with the study of the coverage and overlap between the two bases considered. In the last section, the obtained conclusions are exposed and the limitations of the investigation are mentioned.

## 2. The Concept of Active Ageing

The concept of Active Ageing did not arise spontaneously, but had its roots in the socio-gerontological literature of the 40s and 50s, where the importance of an active lifestyle in old age is emphasized in order to achieve personal satisfaction and which would reach its highest point with the activity theory [31] in force to this day [32].

The term Active Ageing is the result of the evolution of a series of preliminary concepts of the positive paradigm of ageing: Successful Ageing, Productive Ageing, or Healthy Ageing [33,34], which have been used in the same way by experts and scholars of the subject [35]. According to Fernández-Ballesteros [36], these concepts have partially overlapping meanings and partly interchangeable uses.

Regarding the first concept of “Successful Ageing”, it is defined as a positive ageing experience characterized by activity and financial success [37]. According to the definition given by Rowe & Kahn [38], successful ageing means ageing with a low risk of illness and disability, a high mental and physical function and active participation in life. However, for Minkler & Fadem [39], the concept gives rise to many doubts, stating that this definition stigmatizes people with some type of illness or physical or mental dysfunction. “Productive Ageing” is understood as any activity carried out by an older person that contributes to producing goods and services or develops the capacity to produce them, regardless of whether they are paid for the activity or not [40]. Walker [41] states that this definition of productive ageing is hostile, since its main concern regarding the elderly is their economic capacity.

Finally, the term “Healthy Ageing” refers to the process of optimizing opportunities for physical, social and mental health, enabling older people to participate actively and without discrimination in society, while enjoying an independent and good quality of life [35]. Similarly, Hansen-Kyle [42] defines healthy ageing as an elastic adaptation process of a person to his physical and cognitive deceleration to function and participate optimally in all areas of life (physical, cognitive, social, and spiritual). This definition does not seem to consider people with limited resources, who cannot afford the acquisition of quality goods and services that facilitate healthy ageing.

Thus, in the Active Ageing definitions collected in the scientific literature, the influence of these three concepts is detected in order to overcome the limitations of the “productive”, “healthy”, or “successful” vision, expanding its scope of influence to essential dimensions such as participation in social, economic, cultural, spiritual, and civic issues [43].

Although the relevance of the Active Ageing concept in the scientific literature was scarce until 2009 compared to other concepts such as Successful Ageing or quality of life [44], its use became more common since then. Active ageing includes activity and participation together with health, independence and good ageing [45]. The adjective “active” refers both to activity and capacity-building to be a protagonist of one’s own life and its ageing, both aspects being closely related [46]. The human being is an active agent of his own ageing, evolving throughout life in interaction with a world that is also active and through a continuous and dynamic process [47].

Despite its conceptual extent, we frequently find ourselves with a one-dimensional approach to it, focused mainly on a single aspect; either on the economic aspect, referring to the extension of working life, or on physical activity, as a way to improve health in old age, thus reflecting the concepts of Healthy Ageing and Productive Ageing mentioned previously [48]. These partial conceptualizations contradict the intention and recommendations of the WHO and involve the return of the Active Ageing concept to its precursors, so all the effort to create an inclusive concept would be worthless [32].

According to Kalache & Kickbusch [49], the concept of Active Ageing expresses a more inclusive approach that incorporates factors different to health care and which affect the ageing of individuals and populations. An association between activity and opportunities to be healthy during old age is established, highlighting the need to create and maintain opportunities for older people to keep active [50].

Subsequently, in 2002 the WHO adopted this concept, recognizing other factors that affect the ageing process beyond health, such as activity and participation at numerous levels. Thus, it defines it as “the process of optimizing health, participation and security opportunities to improve people’s quality of life as they get older [43] (p. 75). It allows people to fulfil their physical, social and mental well-being potential throughout their life cycle and participate in society according to their needs, desires and abilities, while providing them with protection, security and proper care when they need assistance” [43] (p. 49). Thus, the active ageing concept is currently based on four main pillars: participation, health, security and lifelong learning.

In this way, this “Active Ageing” concept goes beyond and includes concepts such as healthy ageing or productive ageing. Regarding the four pillars, the “Health” concept refers to the right to seek and access medical care and health promotion information. Risk factors, both environmental and behavioral, in chronic diseases and functional decline remain low, while protection factors remain high. People enjoy a greater quantity and quality of life, they stay healthy and can have control of their own lives in old age, therefore, spending less on medical care and treatments.

On the other hand, the term “Participation” implies physical, social, economic, cultural, cognitive, and spiritual activities in interaction with the environment. Support for participation in socio-economic, cultural, and spiritual activities in accordance with their basic human rights, abilities, needs, and preferences through social policies and programmers of the labor market, employment, education, and health, with the elderly contributing productively to society through both paid and unpaid activities as they get older. The term “Security” means security and protection; having basic nutrition, housing, and access to essential services that improve living standards, and meeting social, financial and physical security needs and people’s rights as they get older. Families and communities receive support in their efforts to care for their older members. Finally, “Lifelong Learning” is necessary to invest in training processes aimed at strengthening skills and capabilities to improve the autonomy of the elderly.

In short, people can develop their full potential of physical, social, and mental well-being throughout their life cycle and participate in society according to their needs, desires, and abilities, while they are provided with adequate protection, security, and care when they need assistance [43].

Despite its expansion and importance, in the last decade there are still many studies that point the need to establish an operational definition, universally accepted on the concept of Active Ageing. [51], its definition lacks consensus and a detailed examination of the pillars makes its implementation move away from reality. Thus, Holstein & Minkler [52] consider the active ageing concept mere idealism, whose implementation means imposing values that ignore the reality of ageing and only delves into the positive characteristics of old age. On the other hand, São José & Teixeira [53] raise a similar concern by stating that the productive concept can lead to discrimination based on age, moralism and political ethnocentrism.

However, the active ageing concept has an increasingly important role not only in research, but also in policies and society, largely due to the multidimensional model of the World Health Organization [43], which has been adopted by the European Commission and other bodies [32]. Nevertheless, while the active ageing policy of the WHO considers healthy lifestyles in its conceptualization, the EC focuses more on the contribution of elderly people to society in terms of productive activity after retirement and lifelong learning [54]. However, the WHO has recently reiterated the conceptual validity of the active ageing concept as a response aimed at addressing longevity challenges from individual, local, national and global points of view [55].

Several debates in research on active ageing have emerged. The first one reflects, as already mentioned, the lack of agreement on its definition [32] and the confusion about its components and determinants [56]. A second debate refers to the inclusion of leisure activities in the active ageing concept [57]. Faced with traditional research that considers only productive activities, both paid and unpaid, that create social value [58], authors who criticize are back to the Productive Ageing concept [48] and incorporate leisure activities, considering that cognitive and physical states contribute significantly [59]. In the third place, we can talk about the debate on the dichotomy between active and passive activities [48]. In general, only active leisure activities are considered important for active ageing [60]. However, many older people consider that "normal" leisure activities based on the home and family, generally classified as passive, are more representative of their participation in life [61]. 

## 3. Methodology 

In this section, we describe the work methodology followed in the bibliometric analysis of the scientific production on active ageing, as well as, in the study of overlap and coverage of the databases used, ISI Web of Science (WoS) of Thomson Reuters and Scopus of Elsevier. Prichard [62] states that the application of bibliometric analysis to discipline allows, on the one hand, estimating the degree of consolidation and development of it and addressing the situation in a scientific field in a given situation. In this sense, bibliometric analysis allows the study of the nature and the course of a discipline [62], applying statistical methods [63].

The contribution of this study is to offer information of interest to researchers and professionals relative to the performance of scientific activity: the temporary evolution of total number of publications, the most prolific authors, geographic affiliation of the authors, and journals with greater numbers of studies. Other indicators, such as the number of citations, citations/papers ratio [64] or the h index that allows to evaluate the production an author-level, are a good measure of the researcher’s influence [65].

According to Rueda et al. [66] the bibliometric analysis is limited by three factors: the availability, relevance, and reliability of the information. Therefore, it is very important to choose the database more appropriate for the investigation [67] in order to ensure the validity of the results obtained. Norris and Oppenheim [68] consider the coverage of the database of the area under study as the main factor. Taking into account the above, two of the main international databases, ISI Web of Science (WoS) and Scopus [69] are selected. Both are recognized for their international scope and multidisciplinary, extensive coverage of the scientific literature and of journals. In the case of WoS, its interface allows access to several databases simultaneously: Science Citation Index Expanded (SCI-EXPANDED), Social Sciences Citation Index (SSCI) Arts & Humanities Citation Index (A&HCI), Emerging Sources Citation Index (ESCI).

### 3.1. Method

**Figure 2:**
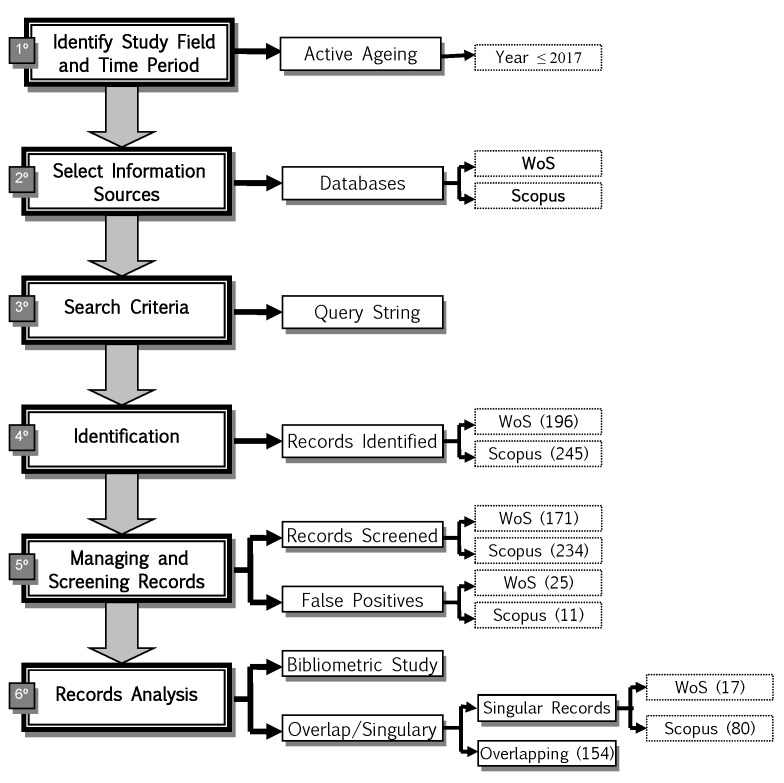
Methodological Procedure. Source: Authors.

In Figure 2, the procedure followed to identify the scientific production on Active Ageing is described. In the first place, the search period was limited to the period ≤ 2017, then the databases (WoS and Scopus) in which the scientific production is indexed were selected. Databases that allow researchers to access different scientific documents (article, article or review, review, article in press, book or book chapter, conference paper, letter, etc.) in all fields of science [69]. To finish, the search equations of terms were elaborated taking into account that each database has its own search system (see Table 1). The searches were limited to articles published in scientific journals because they constitute a representative sample of international scientific activity [70], it was not possible to use the different scientific documents available since both databases do not classify or cover the documents symmetrically.

The problem of which term to use arises in the process of elaboration of the search equations. In recent decades, as seen, researchers in the field of ageing have introduced and used various terms (Productive Ageing, Healthy Ageing, and Active Ageing). Some authors use these terms interchangeably [36], while others distinguish between different meanings [33]. The three concepts share certain characteristics, such as a positive conception of ageing [55]. However, the construction of Active Ageing was formulated to convey a broader concept than that given by the other two terms [71], since it has a multidimensional nature and includes elements related to physical, mental, and social well-being [72], as well as the idea of the productivity capacity of the elderly for society [40], and where activity is considered a broad domain represented by participation in social, economic, cultural, physical, or daily activities [43]. Therefore, all significant activities that improve the well-being of individuals, families, communities, and society in general would be included within the Active Ageing concept [33]. In the same way, Active Ageing has been the concept chosen at political levels for the promotion of the good ageing paradigm. The European Union [73] or the WHO have adopted the Active Ageing concept as a guide for designing ageing policies [55].

The search process allowed to extract the scientific research on this scientific area and to elaborate the ad hoc database to which the bibliometric indicators were applied. The overlap and uniqueness of both databases are also studied. As shown in Figure 2, 196 articles were extracted from the WoS database and 245 from Scopus, which were subjected to a review process with the aim of eliminating those that were irrelevant to the investigation. Thus, the final result was 171 documents published in WoS and 234 in Scopus. The articles were processed with the Refworks bibliographic reference manager.

Finally, in this bibliometric analysis, the following significant indicators from a wide range collected in the Bibliometrics methodology were selected to measure the bibliographic material [74]: productivity indicators in terms of the number of publications and quality indicators that measure the influence of an author through the impact of a publication in relation to the number of citations [75]. The study also uses the h index to combine both concepts [64].

Before starting the process of applying the indicators and analysis, the database obtained is normalized, due to the lack of homogeneity in the process of indexing scientific documents. For example, the specific case of the names of the authors, one of the main problems, was chosen following Spink et al. [76]—the criterion of the coincidence in the ascription of the institutional signature associated with the different variants of the names and surnames.

### 3.2. Methodology of Calculations of Overlap of Web of Science and Scopus

This study analyses overlapping between WoS and Scopus bases and its singularity in the Active Ageing research area following three indicators: Meyer’s Index, traditional overlapping (TO), and relative overlap [77,78].

Meyer’s Index [79] or the “relative index of singularity or peculiarity”, allows comparison of coverage that a database performs on a given topic. The result is interpreted as the degree to which the database covers a certain scientific area [78] and the higher the value of the index, the greater the singularity of the database, which means that it contains a greater number of unique documents [77]. With this criterion, the unique primary sources, contained in a database, are those of greater weight or value (weigh = 1), weight which will be progressively reduced for duplicate documents, weight = 0.5 and triplicates, weight = 0.3.

Meyer’s Index = ∑Number of Documents × Weight/Total Number of Documents Retrieved(1)

To measure the % of overlap between two bases two indicators are used: traditional overlapping (TO) or traditional overlap of coverage between two secondary sources, defined by Gluck [80] and the relative developed by Bearman & Kunberger [81] but defined later by Gluck [80] and cited by Hood and Wilson [82]. The relative overlap allows calculating the overlap of one database in another based on the number of overlapped documents with respect to those of unique presence. The result would be interpreted as the percentage of documents that a base covers of the other. The general formula is as follows:

% Overlap in A = 100 × (|AᑎB|/|A|)(2)

With respect to TO, the higher the percentage, the greater the degree of similarity between the bases. For example, if the result is a coefficient of 0.4 it means 40% similarity or that it is the same as a 60% difference.

% Overlap (TO) = 100 × (|AᑎB|/|AᑌB|)(3)

## 4. Results and Discussion

### 4.1. Temporal Evolution of Publications and Number of Citations

It is observed in Table 2 the temporal distribution of documents on Active Ageing (1987–2017). The period between 2009 and 2017 stands out because it has the largest production of articles, despite not having reached its full potential of citations, which shows the interest that the study of the welfare of the elderly has aroused among researchers in the last 10 years. Within this time interval, 45.6% of the WoS articles and 37.6% of Scopus articles have been published in the last three years.

**Figure 3:**
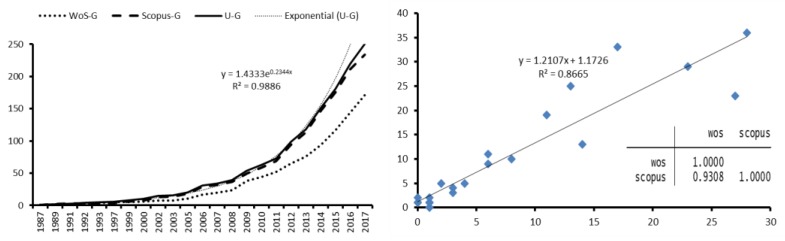
Temporal evolution and Correlation of the articles published on Active Ageing.

If the growth in the publication of articles is analyzed, three periods or phases are observed. There is a first period in which new publications are scarce; these are the so-called Precursors to according to Price’s law of exponential growth [83]. In 2009 a second stage of Exponential Growth starts, in which studies on Active Ageing become the forefront of research (Figure 3). Considering that in the last three years a high level of production is maintained in both bases, it is expected that this trend will continue for the next few years before moving on to the last phase called linear growth where the growth rate is constant and independent of the size of the system. In this phase, growth slows down and the main aim of publications is reviewing.

In Figure 3, a strong correlation is observed between WoS and Scopus with respect to the number of articles collected per year (*R*^2^ = 0.8665). The growth curves separate from each other from the year 2000 onwards.

### 4.2. Growth in the Number of Citations

**Figure 4:**
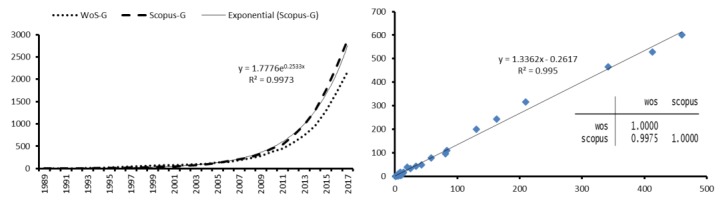
Temporal evolution and Correlation of citations received per year.

The 171 WoS articles received 2,170 citations with a citations/article ratio of 12.69. In terms of the *h*-index=24, 24 studies of the total (171) had 24 citations or more. On the other hand, the 234 articles in the Scopus database received a total of 2,892 citations what represents an average of 12.36 citations/article. The *h*-index = 26, practically identical to that of WoS. Regarding the correlation between the number of citations per year (Figure 4), a strong relationship between both databases is observed with *R*^2^ = 0.995.

Similarly, the growth in the number of citations that the articles received in both databases is constant throughout the period analyzed (year ≤ 2017). The citations reaching its highest level in 2000 (256 citations in WoS and 307 in Scopus) (Figure 4). Specifically, 50.88% (87) of WoS articles and 44.02% (103) of Scopus get between 1–9 and 19.30% (33) and 23.50% (55) between 10–49 citations and 5.85% (10) and 4.70% (11) between 50–100 citations. Also, 21.64% (37) of WoS articles and 25.64% (60) of Scopus do not receive any citations. Only 2.34% (4) of WoS articles and 2.14% (5) of Scopus get 100 citations or more. An important aspect to consider is the one exposed by Merigó et al., [84] (2646), “it must be taken into account that articles published in the last 10 years have not yet shown their maximum level of citations and that access to the first studies is not always available to everybody”.

Of the total number of references selected, the ones with the largest number of citations received (Table 3) are as follows: Busy bodies: Activity, ageing, and the management of everyday life [85] with 245 citations in WoS and 306 in Scopus; Neighborhood design and active ageing [86] with 140 and 163 citations, respectively; and Flow Limitation and Regulation of Functional Residual Capacity during Exercise in a Physically Active Aging Population [87] with 101 and 98.

The article “A strategy for active ageing” [33], which in Scopus is ranked 2nd in the ranking of the most cited, is not indexed in WoS.

### 4.3. Indicators of Overlapping and Singularity of Databases

As previously mentioned, regarding Active Ageing, 171 articles and 234 were located in WoS and Scopus, of which 154 are common to both databases, that is, 90.06% of the documents of WoS and 65.81% of Scopus are overlapping. Thus, 17 (9.94%) and 80 (34.19%) articles, respectively, are single documents collected in a single base (Table 4). With regard to journals, 108 are common (89.99%, Scopus and 68.35% in WoS) and 12 single journals in WoS and 50 in Scopus are identified.

The % of traditional overlap (TO) of the articles between WoS and Scopus is 61.35%. Therefore, between both databases there is a 61.35% similarity, or a 38.65% disparity in relation to articles on Active Ageing. The relative overlap [85] of WoS with respect to Scopus, is 90.06%, and of Scopus is 65.81%, that is, approximately 24% less compared to the overlap of WoS. That is, Scopus covers 90.06% of WoS articles on Active Ageing.

The Meyer’s Index [79] was applied in order to analyze the singularity or relative index of peculiarity of the databases. Table 4 shows the results. A greater singularity of the Scopus database is observed; the Meyer’s index is of 0.67 (articles) and 0.66 (journals) and with 34.19% of single articles and 31.65% of single journals.

There are many researchers who focused on comparing both databases in the last decade examining mainly in their coverage and analysis of the general characteristics [68,93,94,95,96,97,98,99,100,101,102,103,104,105,106]. Considering these investigations, it can be affirmed that these results are corroborated by other studies. For instance, Gavel & Iselid [100] analyzed the journal coverage overlap between both databases with the result of 54% of active titles in Scopus were also in WoS and 84% of active titles in WoS were in Scopus. López-Illescas et al., [102] states that the WoS and Scopus databases differ in terms of scope, data volume, and coverage policies, however, the data obtained is extremely correlated. Affirmation later supported by the study of Archambault et al. [106] for the field of oncology. These authors also found a high correlation relating to the number of articles and citations and concluding that “the two databases offer robust tools for measuring science at the country level” [106] (p. 1325).

### 4.4. Authors

Buys, L. with a total of nine different articles between both bases is the most productive author, followed by Walker, A. with seven authorships and Fernández-Ballesteros, R. with five (Table 5). It is precisely Walker, A., who has the best citations/article average, with seven articles receiving a total of 187 citations and an average of 46.75 in WoS and 417 and 59.57 in Scopus. Following the Law of Exponential Growth enunciated by Lotka [107] on the distribution of authors according to their productivity there is no author with 10 or more articles (large producer). Medium producers, with between one and nine authorships, make up 11.30% (79) and the rest, 88.70% (620), are temporary, that is, with a single authorship. The Productivity Index stands at 1.17.

Among the indicators related with authorship (Table 6), note the collaboration index and number of authorships per article. This index with a value of 3.26 indicates that the average number of authors who sign articles is three, so the majority of articles have multiple authorship. Only 24.30% (61) of the articles are signed by one author. Another indicator analysed was 88.7 Transiency Index with calculation methodology = (Number of authors with a single article published/Total number of authors) × 100; Degree of Collaboration [108] with 75.69 and 1.17 Productivity Index (Number of authorships/Number of authors).

Following the analysis, Table 6 shows the top 10 countries of affiliation of the authors. In the scientific production of articles related to Active Ageing, the United Kingdom with 17.5% (30) articles and 17.1% (40) in WoS and Scopus, ranks first. In addition, this country receives the highest number of citations (571, 905 respectively) and the highest h index (13, 15). The second and third places are occupied by the United States and Spain as the countries with the highest number of authorships.

### 4.5. Journals

**Figure 5:**
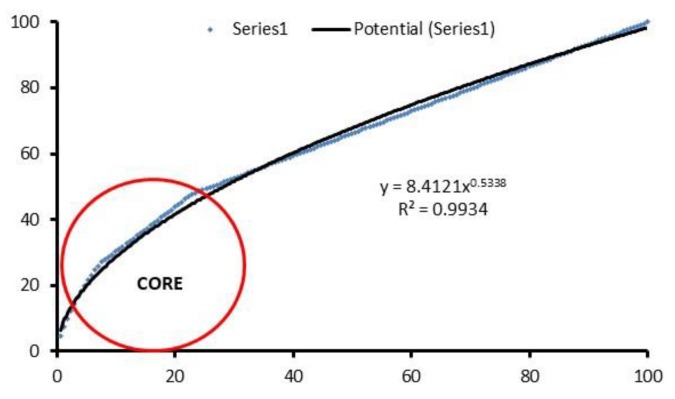
Bradford core of the most productive journals.

The Law of Bradford [109], also known as the scattering law of Bradford’s scientific literature, tries to show that there is a highly unequal distribution of the articles published in journals. Thus, most articles are concentrated in a small population of journals, while a small proportion of articles is dispersed in a large number of journals. Therefore, this law allows identification of the most used journals by researchers for the dissemination of their studies. In Figure 5 the Minimum Bradford Zone (MBZ) is observed, defined by Spinak [110], that shows the number of articles equal to half the number of journals that produce a single article. So, the Bradford Core is made up of those journals whose sum of articles was equal to 66. In this bibliometric analysis, the MBZ is constituted by the first 13 journals with a total of 68 published articles, highlighting Ageing and Society with 12 studies followed by Studies in Health Technology and Informatics with seven, the latter only indexed in Scopus.

Table 7 shows the Ranking of the most productive journals; 131 (77.06%) journals of the total of 170 indexed published a single article and only seven publications published five or more.

The analysis of the thematic areas in which the databases classify the journals shows that there is no clear correspondence between the subject areas in both databases. The areas related to medicine and the elderly stand out, such as geriatrics and gerontology in WoS (42.69% of articles) or medicine and nursing in Scopus (70.51%). By bases, in addition to those already mentioned, note the area of Public Environmental Occupational Health in WoS, with only 16 articles (9.36%), which receives 350 citations, occupying the second place (Table 8), only behind Gerontology. On the other hand, Social Sciences is ranked second in Scopus with 108 articles (46.15%) and 1840 citations, which makes it the area that gets the most citations, even ahead of Medicine, that collects a great number of articles.

## 5. Conclusions

In accordance with the results obtained, and as a conclusion, this section provides a series of essential ideas about research related to the area of Active Ageing (its volume, evolution, visibility, and structure), that can be very useful for future studies, at the same time as comparing the coverage and overlap that two of the main existing databases in the market, WoS and Scopus make on this particular.

After an uncertain start with scarce publications, in 2009, a second phase called “Exponential Growth” began in relation to the production of articles within the area of knowledge on Active Ageing. In this phase not only did the scientific literature increase exponentially, but also the number of researchers, concentrating between 2009 and 2017 85% of the published articles, corroborating the great interest aroused in recent years by everything related to the elderly and their welfare. In the same way, the growth in the number of citations that publications received in those years is constant, reaching its highest level between 2009 and 2014. It is important to clarify that at this point we find a limitation in the analysis of the documents indexed in the database in the period from 1987 to 2009, since was from 2009 that researchers began to use the term “Active Ageing” in their publications and not others like “Successful Ageing or Healthy Ageing”. On the other hand, it is from 2002, as already mentioned, that WHO provides a definition on the concept of Active Ageing that incorporates the approaches of the previous concepts.

As mentioned in the results section, both databases present a strong correlation with respect to the number of articles published annually, as well as in the number of citations received by the articles. These results show what was affirmed by Archambault et al. [106] (p.1325) “the two databases offer robust tools for measuring science”. However, in this research work and in this specific thematic area, the Scopus database collects a greater number of works and receives a greater number of citations. Osca-Lluch et al., [111] (p.1026) states that “Comparisons in order to determine which source is most appropriate for bibliometric studies have not been conclusive, since the relative advantages of one of them with respect to the other depend to a large extent on the discipline and the period of analysis”. There are many studies conducted in this regard [102,103,112,113,114,115,116,117,118,119].

However, and despite these and other similarities, at the same time, there are remarkable differences to those found in relation to the coverage that both databases perform in the Active Ageing area. In the analysis of the singularity of the databases, it is observed that Scopus presents a greater degree of singularity, 34.19% of the total are unique documents versus 9.94% in WoS. Therefore, it is positioned as the base with the highest coverage, in addition to the highest percentage of unique documents, overlaps 90% of the WoS articles.

Based on the precepts developed by Lotka [107] on the distribution of authors according to their productivity, more than 90% are temporary authors having single authorship. The average productivity index per author to be close to 1. L. Buys lead the ranking of the most productive authors with nine articles, followed by A. Walker and R. Fernández-Ballesteros, the only ones with five articles or more. Although there are a large number of affiliation countries, which would demonstrate how geographically widespread the study on Active Ageing is, three countries stand out at the forefront of research: United States, Spain, and the United Kingdom. It is precisely the authors of the UK, who have the best rating, as they obtain a higher h index result (15). Considering the collaboration index expressed as the average number of authors per article, it is above three. This shows that articles with multiple signatures represented more than three quarters of the total.

To end this section of conclusions and in relation to the journals where articles are published, the core of the main journals that collect articles on Active Ageing (core of Bradford) is made up of a total of 13 journals, with Ageing and Society standing out due to the number of articles collected (12). Taking into account the number of citations received, the Journal of Aging Studies would lead the ranking.

Medicine as a general research field includes the highest percentage of articles in both databases. This result is corroborated by the report prepared by the General Foundation CSIC 2016 [120] on “Research on ageing” in the world. In this sense, the report states that more than 90% of scientific publications on ageing address aspects related to biology and medicine. However, given the multidisciplinary nature of studies on the elderly, other areas such as those related to Social Sciences must also be mentioned; although it collects a smaller number of articles behind medicine in this research, it is an area that receives a greater number of citations, which demonstrates the interest in this subject from other areas of knowledge, other than medicine. In the FGCSIC report [120], it is mentioned that the area of Social Sciences as in this research, focused only on active ageing, is the second category with the highest number of publications.

Undoubtedly, the results obtained in this research show that there are many fields in which research on active ageing is very scarce and new, but taking into account the volume of citations in the field of Social Sciences, it is a subject of great interest and with great research potential. Taking into account the report and this research, the research areas with great potential but with little research are those related to studies from the approach of the business and economics area, the educational area and educational research, and the area of technology and physical sciences.

In the same line, in the document developed from the debates of the Congress “Ageing. Research in Spain and Europe” [121] (pp. 6–7), the importance of research on active and healthy ageing is emphasized, focusing on three major areas of interest. The biophysical area involves research on key habits that provide good functional status and longevity, such as nutrition and physical activity. Research on the socio-emotional character of healthy ageing incorporating psychological and social aspects (social, economic and cultural needs, pensions and economic stability, social and family support, etc.). Finally, there is the scope related to cognitive functioning: analyzing social, residential, and geographical contexts (rural-urban) and ageing environments.

Finally, in this research we agree with the perspective collected in the FGCSIC report [120] (p. 2) “the generation of knowledge related to the different facets of ageing of people and societies is essential to contribute to providing solid bases for decision-making processes, with the most integral perspective possible. Beyond prolonging longevity, the challenge is how to age healthily. At a global level, the biggest challenge is to support our elders so that healthy ageing is generalized and takes place in better conditions of personal independence, while facilitating them to continue contributing value to society”. 

The four main limitations of the study are choosing a specific database, the group of bibliometric indicators and techniques chosen for the analysis, a specific search equation (database search strategy), and limiting the unit of analysis to the article, not considering other documents such as books, book articles, congress papers, etc. With regard to the database, only the databases have been considered Web of Science and Scopus, therefore, relevant research not indexed in these two databases can be left out of this overview of active ageing research. 

## Figures and Tables

**Table 1:** Search strategy.

Search Word	Active Aging; Active Ageing
Category	Title
Subject area	All
Document type	Journal article
Period time	Year of publication ≤ 2017
Language	English
Query String	WoS: (TI = (“activ* aging*”) OR TI = (“activ* ageing*”)) AND LANGUAGE: (English) AND Types of documents: (Article) Refined by: Database = (WOS) Period of time: 1900–2017. Scopus: (TITLE (“activ* aging”) OR TITLE (“activ* ageing*”)) AND DOCTYPE (ar) AND PUBYEAR < 2018 AND ( LIMIT-TO (LANGUAGE,“English”))
Search Date	June 2018

Source: Authors.

**Table 2:** Articles, Growth and number of citations for years on Active Ageing in the WoS and Scopus databases.

Year	WoS						Scopus						WoS U Scopus		
	f	hi%	G	TC	$\bar{x}$	*h*-Index	f	hi%	G	TC	$\bar{x}$	*h*-Index	f	hi%	G
1987	1	0.58%	1	39	39.0	1	1	0.43%	1	39	39.0	1	1	0.40%	1
1989	0	0.00%	1	—	—	—	1	0.43%	2	0	0.0	0	1	0.40%	2
1991	1	0.58%	2	103	103.0	1	1	0.43%	3	98	98.0	1	1	0.40%	3
1992	1	0.58%	3	56	56.0	1	0	0.00%	3	—	—	—	1	0.40%	4
1993	1	0.58%	4	2	2.0	1	0	0.00%	3	—	—	—	1	0.40%	5
1997	1	0.58%	5	3	3.0	1	1	0.43%	4	16	16.0	1	1	0.40%	6
1999	0	0.00%	5	—	—	—	2	0.85%	6	14	7.0	1	2	0.80%	8
2000	1	0.58%	6	256	256.0	1	2	0.85%	8	307	153.5	1	2	0.80%	10
2002	2	1.17%	8	73	36.5	2	5	2.14%	13	288	57.6	5	4	1.59%	14
2003	0	0.00%	8	—	—	—	1	0.43%	14	30	30.0	1	1	0.40%	15
2005	3	1.75%	11	97	32.3	3	4	1.71%	18	118	29.5	4	4	1.59%	19
2006	6	3.51%	17	212	35.3	3	11	4.70%	29	256	23.3	5	11	4.38%	30
2007	3	1.75%	20	63	21.0	3	3	1.28%	32	81	27.0	3	3	1.20%	33
2008	4	2.34%	24	114	28.5	3	5	2.14%	37	119	23.8	4	5	1.99%	38
2009	14	8.19%	38	186	13.3	7	13	5.56%	50	217	16.7	8	16	6.37%	54
2010	6	3.51%	44	162	27.0	6	9	3.85%	59	194	21.6	7	9	3.59%	63
2011	8	4.68%	52	152	19.0	6	10	4.27%	69	195	19.5	8	10	3.98%	73
2012	13	7.60%	65	170	13.1	6	25	10.68%	94	301	12.0	9	26	10.36%	99
2013	11	6.43%	76	176	16.0	5	19	8.12%	113	237	12.5	6	19	7.57%	118
2014	17	9.94%	93	162	9.5	6	33	14.10%	146	206	6.2	8	32	12.75%	150
2015	23	13.45%	116	101	4.4	6	29	12.39%	175	125	4.3	6	30	11.95%	180
2016	28	16.37%	144	38	1.4	3	36	15.38%	211	46	1.3	3	39	15.54%	219
2017	27	15.79%	171	5	0.2	1	23	9.83%	234	5	0.2	2	32	12.75%	251
∑	171	100.00%		2170	12.7	24	234	100.00%		2892	12.4	26	251	100.00%	

f = frequency (number of articles published); hi% = relative frequency; G = Growth; TC = total number of citations received for published articles; $\bar{x}$ = Average; *h*-index = Hirsch’s index.

**Table 3:** Ranking of the most cited documents.

Author/s	Year	Title	WoS			Scopus		
			R	TC	C/Y	R	TC	C/Y
Katz, S. [85]	2000	Busy bodies: Activity, aging, and the management of everyday life	1	245	14.4	1	306	18
Michael, Y.L.; Green, M.K.; Farcuhar, S.A. [86]	2006	Neighborhood design and active aging	2	140	12.7	3	163	14.8
Johnson, B.D.; Reddan, W.G.; Seow, K.C.; Dempsey, J.A. [87]	1991	Flow Limitation and Regulation of Functional Residual Capacity during Exercise in a Physically Active Aging Population	3	101	3.9	6	98	3.8
Walker, A. [3]	2008	Commentary: The Emergence and Application of Active Aging in Europe	4	92	11.5	4	111	13.9
Plouffe, L.; Kalache, A. [88]	2010	Towards Global Age-Friendly Cities: Determining Urban Features that Promote Active Aging	5	72	10.3	7	80	11.4
Walker, A.; Maltby, T. [89]	2012	Active ageing: A strategic policy solution to demographic ageing in the European Union	6	68	13.6	5	99	19.8
Bowling, A. [90]	2008	Enhancing later life: How older people perceive active ageing?	7	65	7.2	9	70	7.8
Boudiny, K. [32]	2013	‘Active ageing’: from empty rhetoric to effective policy tool	8	59	14.8	8	72	18
Brown, D.R. [91]	1992	Physical activity, aging, and psychological well-being: An overview of the research.	9	55	2.2	—	—	—
Stenner, P.; McFarquhar, T.; Bowling, A. [46]	2011	Older people and ‘active ageing’: Subjective aspects of ageing actively	10	52	8.7	11	63	9
Walker, A. [33]	2002	A strategy for active ageing	—	—	—	2	173	11.5
Moulaert, T.; Biggs, S. [92]	2013	International and European policy on work and retirement: Reinventing critical perspectives on active ageing and mature subjectivity	12	46	11.5	10	66	16.5

R = rank; TC = the total number of citations received by the published articles; C/Y = average citations received by years.

**Table 4:** Singularity and Meyer’s Index of the databases.

Databases	% Single Documents		Meyer’s Index	
	Articles	Journals	Articles	Journals
WoS	9.94%	10%	0.55	0.57
Scopus	34.19%	31.65%	0.67	0.66

**Table 5:** Ranking of the most productive authors.

R	Name	University	Country	Tf	WoS				Scopus			
					f	TC	C/f	*h*-Index	f	TC	C/f	*h*-Index
1	Buys, L.	Queensland University	Australia	9	6	92	15.33	4	9	119	13.22	5
2	Walker, A.	University of Sheffield	United Kingdom	7	4	187	46.75	4	7	417	59.57	5
3	Fernández-Ballesteros, R	Universidad Autónoma de Madrid	Spain	5	3	9	3.00	1	5	19	3.80	2
4	Aird, R.L.	Queensland University	Australia	4	2	5	2.50	2	4	24	6.00	3
5	Boulton-Lewis, G.M.	Queensland University	Australia	4	4	87	21.75	4	4	95	23.75	4
6	Kalache, A.	Columbia University	United States	4	2	109	54.50	2	4	166	41.50	4

R = rank; Tf = total frequency; f = frequency (number of articles published); TC = the total number of citations received by the published articles; C/f = average citations received by the published articles; *h*-index = Hirsch’s index.

**Table 6:** Top 10 countries of affiliation of the authors.

Country	WoS U Scopus			WoS				Scopus			
	Centers	Authors	Authorships	f	hi%	TC	*h*-Index	f	hi%	TC	*h*-Index
United States	44	94	111	26	15.2%	537	8	32	13.7%	542	10
Spain	29	85	98	23	13.5%	82	6	26	11.1%	108	7
United Kingdom	43	67	79	30	17.5%	571	13	40	17.1%	905	15
Italy	17	65	66	4	2.3%	9	2	17	7.3%	25	2
Germany	15	35	52	13	7.6%	120	7	23	9.8%	141	8
Australia	12	32	51	14	8.2%	182	5	17	7.3%	230	6
Portugal	12	28	36	11	6.4%	36	4	10	4.3%	56	4
Belgium	3	14	23	8	4.7%	117	3	7	3.0%	148	3
Brazil	9	19	23	4	2.3%	21	2	5	2.1%	31	3
Mexico	10	19	22	6	3.5%	15	2	7	3.0%	33	3

f = frequency (number of articles published); hi% = relative frequency; TC = the total number of citations received by the published articles; *h*-index = Hirsch’s index.

**Table 7:** Ranking of the most productive journals.

R	Title	Tf	hi%	WoS			Scopus		
				f	TC	*h*-Index	f	TC	*h*-Index
1	Ageing and Society	12	4.78	12	219	7	12	275	8
2	Studies in Health Technology and Informatics	7	2.79	—	—	—	7	14	2
3	Assistive Technology Research Series	6	2.39	6	7	1	6	14	2
4	Journal of Aging Studies	6	2.39	6	321	4	6	389	4
5	Current Gerontology and Geriatrics Research	5	1.99	—	—	—	5	64	4
6	European Journal of Ageing	5	1.99	5	36	2	5	39	2
7	Journal of Population Ageing	5	1.99	5	1	1	—	—	—

R = rank; Tf = total frequency; hi% = relative frequency; f= frequency (number of articles published); TC = the total number of citations received by the published articles; *h*-index = Hirsch’s index.

**Table 8:** Classification of articles by Subject Area.

WoS						Scopus					
Area	J.	f	TC	C/f	*h*-Index	Area	J.	f	TC	C/f	*h*-Index
Gerontology	24	59	867	14.7	13	Medicine	72	126	1597	12.7	22
Public Environmental Occupation Health	15	16	350	21.9	7	Social Sciences	71	108	1840	17.0	20
Social Sciences Interdisciplinary	8	15	73	4.9	3	Nursing	24	39	428	11.0	10
Geriatric and Gerontology	10	14	180	12.9	5	Engineering	12	27	52	1.9	4
Education Educational Research	7	12	105	8.8	5	Psychology	10	24	463	19.3	11
Social Issues	7	11	41	3.7	3	Arts and Humanities	9	20	374	18.7	10
Social Work	9	11	134	12.2	5	Business Management & Account	13	15	126	8.4	6
Management	6	7	74	10.6	4	Health Professions	6	14	52	3.7	3

J. = journal; f = frequency (number of articles published); TC = the total number of citations received by the published articles; C/f = average citations received by the published articles; *h*-index = Hirsch’s index.

## References

[1] Leon D.A. (2011). Trends in European life expectancy: A salutary view. Int. J. Epidemiol..

[2] United Nations World Population Prospects: The 2017 Revision, Key Findings and Advance Tables. https://www.compassion.com/multimedia/world-population-prospects.pdf.

[3] Walker A. (2008). Commentary: The emergence and application of active aging in Europe. J. Aging Soc. Policy.

[4] Bloom D.E., Canning D. (2008). Global demographic change: Dimensions and economic significance. Popul. Dev. Rev..

[5] European Commission The 2018 Ageing Report: Economic & Budgetary Projections for the EU Member States (2016–2070). https://ec.europa.eu/info/sites/info/files/economy-finance/ip079_en.pdf.

[6] Herskind A.M., McGue M., Holm N.V., Sörensen T.I., Harvald B., Vaupel J.W. (1996). The heritability of human longevity: A population-based study of 2872 Danish twin pairs born 1870–1900. Hum. Genet..

[7] Kaplanis J., Gordon A., Shor T., Weissbrod O., Geiger D., Wahl M., Bhatia G. (2018). Quantitative analysis of population-scale family trees with millions of relatives. Science.

[8] Murabito J.M., Yuan R., Lunetta K.L. (2012). The search for longevity and healthy aging genes: Insights from epidemiological studies and samples of long-lived individuals. J. Gerontol. Series A Biomed. Sci. Med. Sci..

[9] Murtagh B. (2017). Ageing and the social economy. Soc. Enterp. J..

[10] World Health Organization (WHO) (2002). World Population Ageing: 1950–2050.

[11] World Health Organization (WHO) (2015). World Report on Ageing and Health 2015.

[12] United Nations Economic Commission for Europe (UNECE) Introducing the Active Ageing Index. https://ec.europa.eu/eip/ageing/library/policy-brief-introducing-active-ageing-index_en.

[13] Higgins J. (2005). Exploring the politics and policy surrounding senior center gambling activities. J. Aging Stud..

[14] Salari S., Brown B.B., Eaton J. (2006). Conflicts, friendship cliques and territorial displays in senior center environments. J. Aging Stud..

[15] Aalbers T., Baars M.A.E., Olde-Rikkert M.G.M. (2011). Characteristics of effective Internet-mediated interventions to change lifestyle in people aged 50 and older: A systematic review. Ageing Res. Rev..

[16] Heo J., Culp B., Yamada N., Won Y. (2013). Promoting successful aging through competitive sports participation: Insights from older adults. Qual. Health Res..

[17] Boulton-Lewis G.M., Buys L., Lovie-Kitchin J. (2006). Learning and active aging. Educ. Gerontol..

[18] Baker L.A., Cahalin L.P., Gerst K., Burr J.A. (2005). Productive activities and subjective well-being among older adults: The influence of number of activities and time commitment. Soc. Indic. Res..

[19] Principi A., Jensen P.H., Lamura G. (2014). Active Ageing: Voluntary Work by Older People in Europe.

[20] Barnes M., Harrison E., Murray L. (2011). Ageing activists: Who gets involved in older people’s forums?. Ageing Soc..

[21] Petriwskyj A., Warburton J., Everingham J., Cuthill M. (2014). Seniors’ motivations for participation in local governance: Evidence from an Australian study. Local Gov. Stud..

[22] Serrat R., Villar F., Celdrán M. (2015). Factors associated with Spanish older people’s membership in political organizations: The role of active aging activities. Eur. J. Ageing.

[23] Serrat R., Villar F. (2016). Older people’s motivations to engage in political organizations: Evidence from a Catalan study. Int. J. Volunt. Nonprofit Organ..

[24] Serrat R., Villar F., Giuliani M., Zacarés J. (2017). Older people’s participation in political organizations: The role of generativity and its impact on well-being. Educ. Gerontol..

[25] Serrat R., Villar F., Warburton J., Petriwskyj A. (2017). Generativity and political participation in old age: A mixed method study of Spanish elders involved in political organisations. J. Adult Dev..

[26] Burr J.A., Mutchler J.E., Caro F.G. (2007). Productive activity clusters among middle-aged and older adults: Intersecting forms and time commitments. J. Gerontol. Series B Psychol. Sci. Soc. Sci..

[27] Villar F., Celdrán M. (2012). Generativity in older age: A challenge for universities of the third age (U3A). Educ. Gerontol..

[28] Low M.B., MacMillan I.C. (1988). Entrepreneurship: Past research and future challenges. J. Manag..

[29] Tranfield D., Denyer D., Smart P. (2003). Towards a methodology for developing evidence-informed management knowledge by means of systematic review. Br. J. Manag..

[30] Granda-Orive J.I. (2003). Algunas reflexiones y consideraciones sobre el factor de impacto. Arch. de N.a..

[31] Havighurst R.J. (1961). Successful aging. Gerontologist.

[32] Boudiny K. (2013). “Active ageing”: From empty rhetoric to effective policy tool. Ageing Soc..

[33] Walker A. (2002). A strategy for active ageing. Int. Soc. Secur. Rev..

[34] Villar F. (2012). Successful ageing and development: The contribution of generativity in older age. Ageing Soc..

[35] The Swedish National Institute of Public Health European Commission. Healthy Ageing—A Challenge for Europe. http://ec.europa.eu/health/ph_projects/2003/action1/docs/2003_1_26_frep_en.pdf.

[36] Fernández-Ballesteros R. (2008). Active aging. The Contribution Psychology.

[37] Hutchison T., Morrison P., Mikhailovich K. A review of the literature on active ageing. Report prepared for the Australian Government Department of Health 2006. http://www.ub.uib.no/elpub/rokkan/N/N18–03.pdf.

[38] Rowe J.W., Kahn R.L. (1997). Successful aging. Gerontologist.

[39] Minkler M., Fadem P. (2002). Successful ageing: A disability perspective. J. Disabil. Policy Stud..

[40] Caro F.G., Bass S.A., Chen Y.P. (1993). Achieving a Productive Aging Society.

[41] Walker A. (2006). Active ageing in employment: Its meaning and potential. Asia-Pacific Rev..

[42] Hansen-Kyle L. (2005). A concept analysis of healthy aging. Nurs. Forum..

[43] World Health Organization (WHO) (2007). Active Ageing: A policy framework (No. WHO/NMH/NPH/02.8).

[44] Bowling A. (2009). Perceptions of active ageing in Britain: Sivergences between minority ethnic and whole population samples. Age Ageing.

[45] Van Malderen L., Mets T., De Vriendt P., Gorus E. (2013). The Active Ageing-concept translated to the residential long-term care. Qual. Life Res..

[46] Stenner P., McFarquhar T., Bowling A. (2011). Older people and “active ageing”: Subjective aspects of ageing actively. J. Health Psychol..

[47] Fernández-Ballesteros R. (2009). Envejecimiento Activo: Contribuciones de la Psicología.

[48] Boudiny K., Mortelmans D. (2011). A critical perspective: Towards a broader understanding of “active ageing”. Electron. J. Appl. Psychol..

[49] Kalache A., Kickbusch I. (1997). A global strategy for healthy ageing. World Health.

[50] Kalache A. (1999). Active ageing makes the difference. Bull. World Health Organ..

[51] Paúl C., Ribeiro O., Teixeira L. (2012). Active ageing: An empirical approach to the WHO model. Curr. Gerontol. Geriatr. Res..

[52] Holstein M.B., Minkler M., Bernard M., Scharf T. (2007). Critical gerontology: Reflections for the 21st century. Critical Perspectives on Ageing Societies.

[53] São José J.D., Teixeira A.R. (2015). Envelhecimento ativo: Contributo para uma discussão crítica. Análise Soc..

[54] Oxley H. (2009). Policies for healthy ageing: An overview. Organization for Economic Co-Operation and Development (Oecd) Health Working Papers.

[55] Foster L., Walker A. (2015). Active and successful aging: A European policy perspective. Gerontologist.

[56] Tareque M.I., Hoque N., Islam T.M., Kawahara K., Sugawa M. (2013). Relationships between the active aging index and disability-free life expectancy: A case study in the Rajshahi district of Bangladesh. Can. J. Aging.

[57] Marsillas S., De Donder L., Kardol T., Van Regenmortel S., Dury S., Brosens D., Varela J. (2017). Does active ageing contribute to life satisfaction for older people? Testing a new model of active ageing. Eur. J. Ageing.

[58] Rowe J.W., Kahn R.L. (1987). Human aging: Usual and successful. Science.

[59] Silverstein M., Parker M.G. (2002). Leisure activities and quality of life among the oldest old in Sweden. Res. Aging.

[60] Colcombe S., Kramer A.F. (2003). Fitness effects on the cognitive function of older adults: A meta-analytic study. Psychol. Sci..

[61] Clarke A., Warren L. (2007). Hopes, fears and expectations about the future: What do older people’s stories tell us about active ageing?. Ageing Soc..

[62] Pritchard A. (1969). Statistical bibliography or bibliometrics. J. Doc..

[63] Bouyssou D., Marchant T. (2011). Bibliometric rankings of journals based on impact factors: An axiomatic approach. J. Inf..

[64] Hirsch J.E. (2005). An index to quantify an individual’s scientific research output. Proc. Natl. Acad. Sci. USA.

[65] Podsakoff P.M., MacKenzie S.B., Podsakoff N.P., Bachrach D.G. (2008). Scholarly influence in the field of management: A bibliometric analysis of the determinants of university and author impact in the management literature in the past quarter century. J. Manag..

[66] Rueda G., Gerdsri P., Kocaoglu D.F. Bibliometrics and social network analysis of the nanotechnology field. Proceedings of the Portland International Conference on Management of Engineering & Technology (PICMET).

[67] Albort-Morant G., Ribeiro-Soriano D. (2015). A bibliometric analysis of international impact of business incubators. J. Bus. Res..

[68] Norris M., Oppenheim C. (2007). Comparing alternatives to the Web of Science for coverage of the social sciences’ literature. J. Inf..

[69] Bar-Ilan J. (2010). Citations to the ‘Introduction to infometrics’ indexed by WOS, Scopus and Google Scholar. Scientometrics.

[70] Benavides-Velasco C.A., Guzmán-Parra V., Quintana-García C. (2011). Evolución de la literatura sobre empresa familiar como disciplina científica. Cuad. Econ. Dirección Empres..

[71] Foster L., Walker A. (2013). Gender and active ageing in Europe. Eur. J. Ageing.

[72] Peel N., Bartlett H., McClure R. (2004). Healthy ageing: How is it defined and measured?. Australas. J. Ageing.

[73] Hamblin K.A. (2013). Active Ageing in the European Union. Policy Convergence and Divergence.

[74] Ding Y., Rousseau R., Wolfram D. (2014). Measuring Scholarly Impact: Methods and Practice.

[75] Merigó J.M., Blanco-Mesa F., Gil-Lafuente A.M., Yager R.R. (2017). Thirty years of the International Journal of Intelligent Systems: A bibliometric review. Int. J. Intell. Syst..

[76] Spink A., Jansen B.J., Blakely C., Koshman S. (2006). A study of results overlap and uniqueness among major web search engines. Inf. Process. Manag..

[77] Martín-Martín A., Orduna-Malea E., Thelwall M., López-Cózar E.D. (2018). Google Scholar, Web of Science, and Scopus: A systematic comparison of citations in 252 subject categories. J. Inf..

[78] De Granda-Orive J.I., Alonso-Arroyo A., Roig-Vázquez F. (2011). Which data base should we use for our literature analysis?. Arch. N.a..

[79] Meyer D.E., Mehlman D.W., Reeves E.S., Origoni R.B., Evans D., Sellers D.W. (1983). Comparison study of overlap among 21 scientific databases in searching pesticide information. Online Rev..

[80] Gluck M.A. (1990). Review of journal coverage overlap with an extension to the definition of overlap. J. Am. Soc. Inf. Sci..

[81] Bearman T.C., Kunberger W.A. (1977). A Study of Coverage Overlap Among Fourteen Major Science and Technology Abstracting and Indexing Services.

[82] Hood W.W., Wilson C.S. (2003). Informetric studies using databases: Opportunities and challenges. Scientometrics.

[83] Price D.J.S. (1956). The exponential curve of science. Discovery.

[84] Merigó J.M., Mas-Tur A., Roig-Tierno N., Ribeiro-Soriano D. (2015). A bibliometric overview of the Journal of Business Research between 1973 and 2014. J. Bus. Res..

[85] Katz S. (2000). Busy bodies: Activity, aging, and the management of everyday life. J. Aging Stud..

[86] Michael Y.L., Green M.K., Farquhar S.A. (2006). Neighborhood design and active aging. Health Place.

[87] Johnson B.D., Reddan W.G., Pegelow D.F., Seow K.C., Dempsey J.A. (1991). Flow limitation and regulation of functional residual capacity during exercise in a physically active aging population. Am. Rev. Respir. Dis..

[88] Plouffe L., Kalache A. (2010). Towards global age-friendly cities: Determining urban features that promote active aging. J. Urban health.

[89] Walker A., Maltby T. (2012). Active ageing: A strategic policy solution to demographic ageing in the European Union. Int. J. Soc. Welf..

[90] Bowling A. (2008). Enhancing later life: How older people perceive active ageing?. Aging Ment. Health.

[91] Brown D.R. (1992). Physical activity, aging, and psychological well-being: An overview of the research. Can. J. Sport Sci..

[92] Moulaert T., Biggs S. (2013). International and European policy on work and retirement: Reinventing critical perspectives on active ageing and mature subjectivity. Hum. Relations.

[93] Belew R.K. Scientific Impact Quantity and Quality: Analysis of Two Sources of Bibliographic Data. https://arxiv.org/abs/cs/0504036.

[94] Jacsó P. (2005). As we may search: Comparison of major features of the Web of Science, Scopus, and Google Scholar citation-based and citation-enhanced databases. Curr. Sci..

[95] Bakkalbasi N., Bauer K., Glover J., Wang L. (2006). Three options for citation tracking: Google Scholar, Scopus, and Web of Science. Biomed. Digit. Libr..

[96] Bosman J., Van Mourik I., Rasch M., Sieverts E., Verhoeff H. (2006). Scopus Reviewed and Compared: The Coverage and Functionality of the Citation Database Scopus, Including Comparisons with Web of Science and Google Scholar.

[97] Burnham J.F. (2006). Scopus database: A review. Biomed. Digit. Libr..

[98] Meho L.I., Yang K. (2006). Impact of data sources on citation counts and rankings of LIS faculty: Web of Science versus Scopus and Google Scholar. J. Am. Soc. Inf. Sci. Technol..

[99] Falagas M.E., Pitsouni E.I., Malietzis G.A., Pappas G. (2008). Comparison of PubMed, Scopus, Web of Science, and Google Scholar: Strengths and weaknesses. FASEB J..

[100] Gavel Y., Iselid L. (2008). Web of Science and Scopus: A journal title overlap study. Online Inf. Rev..

[101] Meho L.I., Rogers Y. (2008). Citation counting, citation ranking, and h-index of human-computer interaction researchers: A comparison of Scopus and Web of Science. J. Am. Soc. Inf. Sci. Technol..

[102] Lopez-Illescas C., Moya-Anegon F., Moed H.F. (2008). Coverage and citation impact of oncological journals in the Web of Science and Scopus. J. Inf..

[103] Lopez-Illescas C., Moya-Anegon F., Moed H.F. (2009). Comparing bibliometric country-by-country rankings derived from the Web of Science and Scopus: The effect of poorly cited journals in oncology. J. Inf. Sci..

[104] Neuhaus C., Daniel H.D. (2008). Data sources for performing citation analysis: An overview. J. Doc..

[105] Vaughan L., Shaw D. (2008). A new look at evidence of scholarly citation in citation indexes and from Web sources. Scientometrics.

[106] Archambault É., Campbell D., Gingras Y., Lariviére V. (2009). Comparing bibliometric statistics obtained from the Web of Science and Scopus. J. Am. Soc. Inf. Sci. Technol..

[107] Lotka A.J. (1926). The frequency distribution of scientific productivity. J. Acad. Sci..

[108] Subramanyam K. (1983). Bibliometric studies of research collaboration: A review. J. Inf. Sci..

[109] Bradford S.C. (1934). Sources of information on specific subjects. Engineering.

[110] Spinak E. (1996). Diccionario Enciclopédico de Bibliometría, Cienciometría e Informetría.

[111] Osca-Lluch J., Miguel S., González C., Peñaranda-Ortega M., Quiñones-Vidal E. (2013). Coverage and overlap of the Web of Science and Scopus in the analysis of the Spanish scientific activity in Psychology. Ann. Psychol..

[112] Kulkarni A.V., Aziz B., Shams I., Busse J.W. (2009). Comparisons of citations in Web of Science, Scopus, and Google Scholar for articles published in general medical journals. JAMA.

[113] Vieira E.S., Gomes J.A. (2009). A comparison of Scopus and Web of Science for a typical university. Scientometrics.

[114] Wainer J., Xavier E.C., Bezerra F. (2009). Scientific production in computer science: A comparative study of Brazil and other countries. Scientometrics.

[115] Torres-Salinas D., Lopez-Cózar E.D., Jiménez-Contreras E. (2009). Ranking of departments and researchers within a university using two different databases: Web of Science versus Scopus. Scientometrics.

[116] Mingers J., Lipitakis E. (2010). Counting the citations: A comparison of Web of Science and Google Scholar in the field of business and management. Scientometrics.

[117] Franceschet M. (2009). A comparison of bibliometric indicators for computer science scholars and journals on Web of Science and Google Scholar. Scientometrics.

[118] Etxebarria G., Gomez-Uranga M. (2010). Use of Scopus and Google Scholar to measure social sciences production in four major Spanish universities. Scientometrics.

[119] Álvarez-García J., Durán-Sánchez A., del Río-Rama M.D.L.C. (2018). Scientific coverage in community-based tourism: Sustainable tourism and strategy for social development. Sustainability.

[120] FGCSIC (2016). Aging Research Report 2016—FGCSIC.

[121] Rodríguez V.R., Manas L.R., Castiello M.S., Martín R.D. (2012). Ageing: Research in Spain and Europe. Rev. Española Geriatría Gerontol..

